# New insights into the phylogeny of *Sinocarum* (Apiaceae, Apioideae) based on morphological and molecular data

**DOI:** 10.3897/phytokeys.175.60592

**Published:** 2021-03-17

**Authors:** Yan-Ping Xiao, Xian-Lin Guo, Megan Price, Wei Gou, Song-Dong Zhou, Xing-Jin He

**Affiliations:** 1 Key Laboratory of Bio-Resources and Eco-Environment of Ministry of Education, College of Life Sciences, Sichuan University, 610065, Chengdu, Sichuan, China Sichuan University Chengdu China; 2 Sichuan Key Laboratory of Conservation Biology on Endangered Wildlife, College of Life Sciences, Sichuan University, 610065, Chengdu, Sichuan, China Sichuan University Chengdu China

**Keywords:** Apiaceae, morphology, phylogeny, *
Sinocarum
*

## Abstract

*Sinocarum* is a Sino-Himalayan endemic genus of Apiaceae and distributed in high-elevations from Nepal to SW China. In this study, morphological characteristics were combined with nuclear internal transcribed spacer (ITS) and two chloroplast DNA (cpDNA) intron sequences (*rpl16* and *rps16*) to determine the phylogenetic placement of *Sinocarum* and the infrageneric relationships between five *Sinocarum* species. The results confirmed that *Sinocarum* was a polyphyletic group separated into two clades, *Acronema* and East Asia clades. *S. coloratum*, the generic type of *Sinocarum*, *S. cruciatum*, *S. vaginatum* and *S. filicinum* are in the *Acronema* clade. Among them, the first three species are clustered into a subclade and are closely related to the genus *Acronema*. While *S. filicinum* has a close affinity with *Meeboldia*. *S. schizopetalum* did not ally with its congeners we collected and is allied closely with members of the distantly related East Asia clade. In addition, the fruit of the *Acronema* clade *Sinocarum* species is usually oblong-ovoid or ovoid, and the pollen is super-rectangular, while the *Sinocarum* species in the East Asia clade have broad-ovoid fruit and sub-rhomboidal pollen. This study has furnished cumulative evidence to reduce phylogenetic uncertainty and provide a more comprehensive description of the plant morphology, fruit morphology and anatomy, and pollen morphology of these five Chinese *Sinocarum* species.

## Introduction

*Sinocarum* H. Wolff ex R. H. Shan & F. T. Pu (1980: 374) was transferred from the genus *Carum* L. (1753: 263) by Wolff (1927), but formally described by Shan and Pu (1980). *Sinocarum* encompasses about 20 species, with eight species (four endemic) in China and is distributed at high-elevation in the Sino-Himalayan region from Nepal to SW China (Pu et al. 2005). It is usually classified by a suite of characteristics: elongate rhizome, expanded petiole sheaths, obtuse at apex and clawed at base petals and oblong-ovoid fruit (Shan et al. 1980; Pu et al. 2005). Despite several easily recognizable characteristics, there remains morphological and taxonomic confusion in the genus, including the lack of morphological description and specimens of mature fruit, unclear intergeneric boundary and excessive use of synonyms. *Sinocarum* is a taxonomically complex genus that is closely related to *Acronema* Falcon. ex Edgew. (1846: 51) and sometimes difficult to distinguish (Pu et al. 2005). Thus, further work and more extensive specimen collections are needed to clarify the situation.

Palynological study of *Sinocarum* mainly focused on seven species, these being *S. coloratum* (Diels) H. Wolff ex R. H. Shan & F. T. Pu (1985: 33), *S. cruciatum* (Franch.) H. Wolff ex R. H. Shan & F. T. Pu (1985: 33), *S. dolichopodum* (Diels) H. Wolff ex R. H. Shan & F. T. Pu (1985: 38), *S. filicinum* H. Wolff (1929: 182), *S. pauciradiatum* R. H. Shan & F. T. Pu (1980: 374), *S. schizopetalum* (Franch.) H. Wolff ex R. H. Shan & F. T. Pu (1985: 33) and *S. vaginatum* H. Wolff (1929: 183). The seven species were observed through light microscope (LM) and scanning electron microscope (SEM) (Wang 1998; Shu and She 2001). The study indicated that the equatorial view of *Sinocarum* pollen was usually broad-ellipsoidal, and the equatorial exine was rugulate (Shu and She 2001).

Previous studies have shown that fruit characteristics play a key role in the classification of subfamily Apioideae (Kljuykov et al. 2004; Lyskov et al. 2017; Guo et al. 2018; Jia et al. 2019). The fruit characteristics of *Sinocarum* species are described in *Flora Reipublicae Popularis Sinicae* and *Flora of China*. The fruit of *Sinocarum* is oblong-ovoid with 5 filiform ribs, but only young fruit is involved, and the mature fruit is unknown (Shan and Pu 1985; Pu et al. 2005). Hence, the definition of the fruit’s morphological and anatomical characteristics needs to be supplemented to allow for better identification.

Similarly, previous molecular studies have been limited and results ambiguous. *Sinocarum* was found to be polyphyletic based on ITS, cpDNA sequences and limited specimen materials (*S. coloratum*, *S. cruciatum* and *S. dolichopodum*) and there has been no consensus on its phylogenetic placement (Valiejo-Roman et al. 2002; Zhou et al. 2008; Zhou et al. 2009; Downie et al. 2010). Consequently, there is a gap in our understanding of *Sinocarum*’s phylogeny and infrageneric classification due to insufficient specimen sampling. Together with limited definitions of morphological characteristics, there is a need to study the phylogeny and morphology of this genus based on new, comprehensive materials.

Therefore, the objective of this study was to estimate the phylogenetic placement of *Sinocarum* and the infrageneric relationships of the five *Sinocarum* species we collected. This is the first comprehensive phylogenetic analysis of *Sinocarum* using morphology and three DNA regions data (i.e. ITS, *rpl16* and *rps16*). Given this more comprehensive analysis, we also discuss the significance of using morphology in phylogenic analyses. In addition, we provide more comprehensive descriptions for the plant morphology, fruit morphology and anatomy, pollen morphology and identification of herbarium specimens of five accepted *Sinocarum* species. We believed that this study will contribute to a better understanding of the phylogenetic status, infrageneric relationships and morphological identification of *Sinocarum*.

## Materials and methods

### Field investigation, morphology study and specimen examination

Samples were obtained from type localities and adjacent areas of *S. coloratum* (Mt. Yulong, Yunnan), *S. cruciatum* (Mt. Jizu, Yunnan), *S. filicinum* (Mt. Cang; Mt. Jizu, Yunnan), *S. schizopetalum* (Mt. Cang, Yunnan) and *S. vaginatum* (Mt. Cang, Yunnan). The fruit of *Meeboldia yunnanensis* (H. Wolff) Constance & F. T. Pu (1998: 70) was obtained from Kunming, Yunnan. Photographs of specimens were made using a Nikon D5600 camera. Fruits were observed and photographed using a stereomicroscope, Nikon SMZ 25 (Japan), and five representative fruit samples were selected to observe characters and measure their size, and then calculate the average value. Pollen grains from the anthers of specimens were directly mounted on copper stubs with conductive carbon adhesive tabs using a needle, sputtercoated with gold, and observed with a Hitachi-SX-450 SEM (Japan). The continuous section of the middle transection of the mericarp was made by the normal paraffin section method. And the section was observed and photographed using stereomicroscope Nikon SMZ25 (Japan). A total of ten pollen grains were selected to measure their length of polar axis (P) and equatorial axis (E), and calculate their average value, ratio of polar axis to equatorial axis (P/E) and size index ($\sqrt{P\times E}$). The micromorphological characteristics of pollen were described according to Shu and She (2001). Morphological characteristics were measured using Kayotype (Altınordu et al. 2016). Voucher specimens were deposited in the herbarium of Natural History Museum of Sichuan University (SZ) (Table 1).

**Table 1.** Voucher details and GenBank accession numbers of taxa used in this study.

Taxa	Voucher	Locality	ITS	*rpl16*	*rps16*
*Acronema astrantiifolium* H. Wolff	T2010093003(SZ)	Muli, Sichuan, China	KP940757	KP940829	KP940901
*A. muscicola* (Hand.-Mazz.) Hand.-Mazz.	XZ2011081741(SZ)	Xizang, China	KP940756	KP940828	KP940900
*A. paniculatum* (Franch.) H. Wolff	T2010100602(SZ)	Xiangcheng, Sichuan, China	KP940758	KP940830	KP940902
*A. schneideri* H. Wolff	ZJ810826(KUN)	Shangri-La, Yunnan, China	EU236156	FJ385070	–
*Anthriscus sylvestris* (L.) Hoffm.	ZJ0566(KUN)	Daocheng-Litang, Sichuan, China	EU236159	FJ385078	FJ385176
*Chaerophyllum prescottii* DC.	ZJ0744(KUN)	Habahe, Xinjiang, China	FJ385039	FJ385084	FJ385183
*Changium smyrnioides* H. Wolff	J101(KUN)	Jiangsu Institute of Botany, China	DQ517340	FJ385088	FJ385187
*Chuanminshen violaceum* M. L. Sheh & R. H. Shan	J105(KUN)	Xinlong, Sichuan, China	FJ385040	FJ385089	FJ385188
*Cyclorhiza peucedanifolia* (Franch.) Constance	J034(KUN)	Lijiang, Yunnan, China	FJ385042	FJ385092	FJ385191
*C. waltonii* (H. Wolff) M. L. Sheh & R. H. Shan	ZJ0536(KUN)	Derong, Sichuan, China	EU236165	FJ385093	FJ385192
*Ferula kingdon-wardii* H. Wolff	ZJ810846(KUN)	Shangri-La, Yunnan, China	EU236166	FJ385094	FJ385193
*Halosciastrum melanotilingia* (H. Boissieu) Pimenov & V. N. Tikhom.	Pimenov & Kljuykov 200 (MW)	Khasan distr., Primorsk Terr., Russia	AY328937, AY330503	–	–
*Hansenia forbesii* (H. Boissieu) Pimenov et Kljuykov	666939(SZ)	–	GU390407	–	–
*H. weberbaueriana* (Fedde ex H. Wolff) Pimenov et Kljuykov	ZJ0697(KUN)	KIB nursery, Yunnan, China	EU236180	FJ385115	FJ385212
*Harrysmithia franchetii* (M. Hiroe) M. L. Sheh	ZJ0748(KUN)	Luquan, Yunnan, China	FJ385044	FJ385097	FJ385195
*H. heterophylla* H. Wolff	T2012052603 (SZ)	Baoxing, Sichuan, China	KP940763	–	–
*Haplosphaera phaea* Hand.-Mazz.	ZJ0521(KUN)	Shangri-La, Yunnan, China	EU236167	FJ385096	FJ385194
*Heptaptera anisoptera* (DC.) Tutin	Pimenov & Kljuykov 438 (MW)	Lorestan, Iran	AY941273, AY941301	–	–
*Komarovia anisosperma* Korovin	–	–	AF077897	AF094434	AF110555
*Ligusticum delavayi* Franch.	ZJ810841(KUN)	Shangri-La, Yunnan, China	EU236174	FJ385106	FJ385204
*Meeboldia achilleifolia* (DC.) P. K. Mukh. & Constance	Pimenov & Kljuykov 28 (MW)	Langtang National Park, Nepal	AY038206, AY038220	–	–
*M. yunnanensis* (H. Wolff) Constance & F. T. Pu	ZJ0673(KUN)	Fumin, Yunnan, China	EU236178	FJ385110	FJ385208
*Oenanthe hookeri* C. B. Clarke	ZJ0519(KUN)	Shangri-La, Yunnan, China	EU236182	–	–
*Oreocomopsis stelliphora* (Cauwet & Farille) Pimenov & Kljuykov	Farille 81-421 (G)	N Annapurna, Nepal	GQ379322	–	–
*Oreomyrrhis involucrata* Hayata	J111(KUN)	Taiwan, China	FJ385052	–	FJ385218
*Ostericum scaberulum* (Franch.) C. Q. Yuan & R. H. Shan	YL757(KUN)	Lijiang, Yunnan, China	FJ385053	FJ385121	FJ385219
*Pachypleurum xizangense* H. T. Chang & R. H. Shan	Watson & Gilbert 1580 (E, EBH)	Madoi, Qinghai, China,	KJ660841	–	KJ660442
*Physospermopsis cuneata* H. Wolff	J066(KUN)	Lijiang, Yunnan, China	FJ385055	FJ385125	FJ385221
*P. kingdon-wardii* (H. Wolff) C. Norman	ZJ810822(KUN)	Sichuan, China	EU236190	FJ385127	FJ385223
*P. muliensis* R. H. Shan & S. L. Liou	ZJ0686(KUN)	Ninglang, Yunnan, China	EU236191	FJ385128	FJ385224
*P. rubrinervis* (Franch.) C. Norman	FED 378 (E)	Shangri-La, Yunnan, China	AF164836, AF164861	–	–
*P. shaniana* C. Y. Wu & F. T. Pu	ZJ0678(KUN)	Ninglang, Yunnan, China	EU236192	FJ385129	FJ385225
*Pimpinella acuminata* (Edgew.) C. B. Clarke	ZJ0503(KUN)	Lijiang, Yunnan, China	EU236193	FJ385130	FJ385226
*P. henryi* Diels	ZJ0524(KUN)	Shangri-La, Yunnan, China	EU236195	FJ385132	FJ385228
*P. purpurea* (Franch.) H. Boissieu	ZJ0527(KUN)	Shangri-La, Yunnan, China	EU236197	FJ385133	FJ385229
*Pleurospermum franchetianum* Hemsl.	ZJ0573(KUN)	Yajiang-Kangding, Sichuan, China	EU236198	FJ385137	FJ385232
P. hookeri var. thomsonii C. B. Clarke	ZJ0545(KUN)	Sichuan, China	EU236199	FJ385138	FJ385233
*P. wrightianum* H. Boissieu	ZJ0669(KUN)	Shangri-La, Yunnan, China	EU236201	FJ385140	FJ385235
*P. yunnanense* Franch.	ZJ091033(KUN)	Shangri-La, Yunnan, China	EU236202	FJ385141	FJ385236
*Pternopetalum botrychioides* (Dunn) Hand.-Mazz.	ZJ04(KUN)	Suijiang, Yunnan, China	EU236203	FJ385142	FJ385237
*P. cardiocarpum* (Franch.) Hand.-Mazz.	ZJ0581(KUN)	Luding-Mianning, Sichuan, China	EU236204	FJ385143	FJ385238
*P. davidii* Franch.	ZJ06(KUN)	Suijiang, Yunnan, China	EU236205	FJ385144	FJ385239
*Pterygopleurum neurophyllum* (Maxim.) Kitag.	–	–	AY509127	–	–
*Rupiphila tachiroei* (Franch. & Sav.) Pimenov & Lavrova	Pimenov & Kljuykov 169 (MW)	Primorsk Terr., Russia	AY328952, AY330518	–	–
*Sinocarum bellum* (C. B. Clarke) Pimenov & Kljuykov	Skvortzov & Proskurjakova (MHA)	West Bengal, India	MK309872	–	–
*S. coloratum* (Diels) H. Wolff ex R. H. Shan & F. T. Pu	XYP19071901(SZ)	Lijiang, Yunnan, China	MN846685*	MN852960*	MN852964*
*S. coloratum* (Diels) H. Wolff ex R. H. Shan & F. T. Pu	YL561(KUN)	Lijiang, Yunnan, China	FJ385063	FJ385154	FJ385248
*S. coloratum* (Diels) H. Wolff ex R. H. Shan & F. T. Pu	–	–	AY328927	–	–
*S. cruciatum* (Franch.) H. Wolff ex R. H. Shan & F. T. Pu	XYP19080301(SZ)	Dali, Yunnan, China	MN846686*	MN852961*	MN852965*
*S. cruciatum* (Franch.) H. Wolff ex R. H. Shan & F. T. Pu	ZJ0672(KUN)	Shangri-La, Yunnan, China	EU236209	FJ385155	FJ385249
*S. cruciatum* (Franch.) H. Wolff ex R. H. Shan & F. T. Pu	–	–	AY038199, AY038213	–	–
*S. dolichopodum* (Diels) H. Wolff ex R. H. Shan & F. T. Pu	ZJ0548(KUN)	Sichuan, China	EU236208	FJ385156	FJ385250
*S. filicinum* H. Wolff (JZS)	XYP19080302(SZ)	Dali, Yunnan, China	MT586806*	MT588116*	MT588118*
*S. filicinum* H. Wolff (CS)	XYP19091803(SZ)	Dali, Yunnan, China	MT586807*	MT588117*	MT588119*
*S. schizopetalum* (Franch.) H. Wolff ex R. H. Shan & F. T. Pu	XYP19080401(SZ)	Dali, Yunnan, China	MN846687*	MN852962*	MN852966*
*S. vaginatum* H. Wolff	XYP19080402(SZ)	Dali, Yunnan, China	MN846688*	MN852963*	MN852967*
*S. wolffianum* (Fedde ex H. Wolff) P. K. Mukh. & Constance	Pimenov & Kljuykov 62 (MW)	Yumthang, Sikkim, India	MK309871	–	–
*Sinolimprichtia alpina* H. Wolff	0465919(KUN)	Xizang, China	FJ385064	FJ385157	FJ385251
*Sium frigidum* Hand.-Mazz.	ZJ0520(KUN)	Shangri-La, Yunnan, China	EU236210	–	–
*S. ventricosum* (H. Boissieu) Li S. Wang & M. F. Watson	–	–	AY038200, AY038214	–	–
*Spuriopimpinella arguta* (Diels) X. J. He & Z. X. Wang	T2012091505 (SZ)	Songxian, Henan, China	KP940760	–	–
*S. brachycarpa* (Kom.) Kitag.	T2012093001 (SZ)	Anshan, Liaoning, China	KP940761	–	–
*Tilingia ajanensis* Regel & Til.	Pimenov & Kljuykov 139 (MW)	Saghalien, Russia	AY328939, AY330505	–	–
*Torilis japonica* (Houtt.) DC.	ZJ0623(KUN)	Hongyuan, Sichuan, China	EU236214	FJ385163	AF123741
*Tongoloa elata* H. Wolff	Pimenov et al. 180 (MW)	Hongyuan-Barkam, Sichuan, China	AY038207, AY038221	–	–
*T. gracilis* H. Wolff	ZJ0554 (KUN)	Daocheng, Sichuan, China	EU236211	–	–
*T. loloensis* (Franch.) H. Wolff	ZJ0501(KUN)	Lijiang, Yunnan, China	EU236212	FJ385160	FJ385254
*T. silaifolia* (H. Boissieu) H. Wolff	ZJ810821(KUN)	Sichuan, China	EU236213	FJ385161	FJ385255
*T. tenuifolia* H. Wolff	J075(KUN)	Lijiang, Yunnan, China	FJ385066	FJ385162	FJ385256
*Trachydium simplicifolium* W. W. Sm.	J091(KUN)	Lijaing, Yunnan, China	FJ385067	FJ385164	FJ385257
*Vicatia bipinnata* R. H. Shan & F. T. Pu	ZJ0564(KUN)	Daocheng-Litang, Sichuan, China	EU236217	FJ385167	FJ385260

– unavailable sequences. * Newly generated sequences; otherwise, sequences were obtained from GenBank.

The related specimens in A, BM, CDBI, E, GB, GH, HNWP, IBSC, JAY, K, KATH, KUN, NAS, NWFC, NY, P, PE, SZ, USP and W were studied and presented in Table 2. Information and photographs of type specimens were gathered from Tropicos (http://www.tropicos.org), the International Plant Names Index (http://www. ipni.org) and JSTOR Global Plants (http://plants.jstor.org).

**Table 2.** *Sinocarum* specimens examined in this study.

Species	Type specimens	Additional specimens examined
*S. coloratum*	**China. Yunnan**: Grassy ledges of cliffs on the eastern flank of the Lichiang Range, 11–12000 ft, Lat. 27°25'N, September 1906, *G. Forrest 3060* (lectotype: E!, designated by Watson, 1998: 382; isolectotype: BM0000574892).	**China**. Without specific locality, *Lianda expedition 21341* (KUN); without specific locality, *Lianda expedition 21612* (KUN). **Sichuan Province**: Konkaling, Tsungu, 3850 m, 30 August 1937, *T. T. Yu 13026* (PE); Daocheng, Mt. Gongga, 3200 m, 1 September 1981, *Qinghai-Xizang expedition 6025* (KUN); Daocheng, Mt. Gongga, 4500 m, 29 August 1981, *Qinghai-Xizang expedition 5581* (KUN). **Yunnan Province**: Shangri-La, 3500 m, 16 August 1962, *Zhongdian expedition 957* (PE); Lijiang, 3 September 1939, *Z. G. Zhao 30577* (PE); Lijiang, 3000 m, 11 August 1937, *T. T. Yu 15416A* (PE); Binchuan, Ki-chan, 2900 m, 18 September 1929, *R. C. Ching 24714* (PE); Mekong-Yangtze divide, 4000 m, August 1914, *G. Forrest 12970* (PE); Likiang Snow Range, 3 September 1939, *R. C. Ching 30577* (KUN); Fugong, Mt. Biluo, 4300 m, 12 September 1964, *S. G. Wu 8780* (KUN); Fugong, Mt. Biluo, 12 September 1964, *S. G. Wu 8807* (KUN); Shangri-La, Mt. Haba, 31 August 1962, *Zhongdian expedition 1800* (KUN); Shangri-La, Mt. Haba, 8 September 1962, *Zhongdian expedition 1923* (KUN); Lijiang, Mt. Yulong, 3800 m, 19 July 2019, *Y. P. Xiao & Q. P. Jiang XYP19071901* (SZ); Lijiang, Mt. Yulong, 3800 m, 27 September 2019, *Y. P. Xiao & Q. P. Jiang XYP19092701* (SZ).
*S. cruciatum*	**China. Yunnan**: Mt. Ki-chan, 2800 m, 10 September 1884, *Delavay 182* (lectotype: P03224861!, designated by Pimenov, 2017: 219; isolectotypes: P02284823, P03224868).	**China. Yunnan Province**: Binchuan, Mt. Jizu, 3000 m, 3 August 2019, *Y. P. Xiao & Q. P. Jiang XYP19080301* (SZ).
*S. filicinum*	**China. Yunnan**: Eastern flank of the Tali Range, 2540 m, *G. Forrest 6963* (lectotype: E!, designated by M. Farille; isolectotype: K000685663!).	**China**. Without specific locality, *Lianda expedition 12092* (KUN). **Yunnan Province**: Dali, 22 June 1945, *H. C. Wang 4412* (PE); Dali-Hejiang, September 1941, *H. C. Wang 1396* (PE); Binchuan, Mt. Jizu, 3200 m, *R. C. Ching 24920* (PE); Binchuan, Mt. Jizu, *Anonymous 2447* (PE); Binchuan, Mt. Jizu, 3000 m, 3 August 2019, *Y. P. Xiao & Q. P. Jiang XYP19080302* (SZ); Dali, Mt. Cang, 3200 m, 18 September 2019, *Y. P. Xiao & Q. P. Jiang XYP19091803* (SZ).
*S. schizopetalum*	**China. Yunnan**: Mt. Tsang-chan, 4000 m, 25 July 1884, *Delavay 196* (lectotype P!, designated by Pimenov, 2017: 221; isolectotypes: K000685665!, PE).	Without specific locality, *C. Y. Wu & D. Y. Liu 20581* (PE, PEY). **Yunnan Province**: Dali, Mt. Cang, 3800 m, 4 August 2019, *Y. P. Xiao & Q. P. Jiang XYP19080401* (SZ).
*S. vaginatum*	**China. Yunnan**: Mt. Ghi Shan, 11000 ft. (About 3350 m), open pasture, August 1917, *G. Forrest 15484* (lectotype: E!, designated by Pimenov, 2017: 221; isolectotype: K000685664).	**China**. **Sichuan Province**: Yanyuan, Mt. Xiaogao, 2150 m, October 1986, *Z. H. Pan & Y. J. Li & F. T. Pu 964* (CDBI). **Yunnan Province**: Dali, Mt. Cang, 21 August 1944, *H. C. Wang 4511* (PE); Dali, Mt. Cang, 4 August 2019, *Y. P. Xiao & Q. P. Jiang XYP19080402* (SZ).

### DNA extraction, amplification and sequencing

Total genomic DNA was extracted from silica gel-dried leaves and herbarium materials according to the protocols of plant genomic DNA kit (Tiangen Biotech, Beijing, China). Nuclear ribosomal DNA (nrDNA) ITS sequences and two chloroplast DNA (cpDNA) intron sequences (*rpl16* and *rps16*) were applied to phylogenetic analyses. The primers ITS4 (5’-TCC TCC GCT TAT TGA TAT GC-3’) and ITS5 (5’-GGA AGT AAA AGT CGT AAC AAG G-3’; White et al. 1990) were used for PCR-amplification of a complete ITS fragment. The *rpl16* intron region was amplified with primers F71(5’-GCT ATG CTT AGT GTG TGA CTC GTT G-3’) and R1516 (5’-CCC TTC ATT CTT CTA TGT TG-3’; Jordan et al. 1996; Kelchner and Clark 1997). The *rps16* intron was amplified using primers *rps16* 5’exon (5’-AAA CGA TGT GGN AGN AAR CA-3’) and *rps16* 3’exon (5’-CCT GTA GGY TGN GCN CCY TT-3’; Downie and Katz-Downie 1999). Amplification was undertaken in a 30 µL mixture of 2 µL plant total DNA, 10 µL ddH_2_O, 1.5 µL forward primer, 1.5 µL reverse primer and 15 µL 2 × Taq MasterMix (cwbio, Beijing, China). The amplification of the ITS region was obtained by initial denaturation for 3 min at 94 °C, followed by 30 cycles of 45 s at 94 °C, 60 s at 54 °C, and 90 s at 72 °C, and then a final extension of 10 min at 72 °C. The amplification of the *rpl16* region was obtained by initial denaturation for 3 min at 94 °C, followed by 36 cycles of 45 s at 94 °C, 70 s at 58.5 °C, and 90 s at 72 °C, and then a final extension of 10 min at 72 °C. Whereas amplification of *rps16* region was obtained by initial denaturation for 3 min at 94 °C, followed by 36 cycles of 45 s at 94 °C,70 s at 54 °C, and 90 s at 72 °C, and then a final extension of 10 min at 72 °C. All PCR products were separated using a 1.5% (w/v) agarose TAE gel and sent to Sangon (Shanghai, China) for sequencing. New sequences generated for this study have been deposited in GenBank (Table 1).

### Sequence alignment and phylogenetic analysis

We used 53 nrDNAITS sequences obtained from GenBank, and six sequences newly sequenced for this study (Table 1), to infer the phylogenetic placement of *Sinocarum*. Seventy-four accessions obtained from GenBank for the nrDNA (ITS) and cpDNA (*rpl16* and *rps16*), and 15 accessions newly sequenced (Table 1) represented 35 species from 21 genera of Apiaceae and were used to reconstruct the phylogenetic tree of the *Acronema* clade. Tribe Scandiceae was selected as the outgroup (Downie et al. 2000a; Zhou et al. 2008; Zhou et al. 2009). Eighty-three accessions obtained from GenBank for the nrDNA (ITS) and cpDNA (*rpl16* and *rps16*), and three accessions newly sequenced (Table 1) represented 31 species from 15 genera of Apiaceae and were used to reconstruct the phylogenetic tree of the East Asia clade. Tribe Pleurospermeae was selected as the outgroup (Downie et al. 2000b; Zhou et al. 2008; Zhou et al. 2009). Sequence data for the ITS 5.8S region were excluded from the analysis because they were unavailable for several previously published taxa.

SeqMan (Burland 2000) was used to assemble DNA sequences and obtain consensus sequences. DNA sequences were aligned with ClustalX ver. 2.1 (Larkin et al. 2007) and then adjusted manually using MEGA7 (Kumar et al. 2016). Phylogenetic analyses of data were conducted by employing Maximum Likelihood (ML) and Bayesian Inference (BI) methods. Maximum Likelihood phylogenetic reconstruction was performed using RAxML-HPC ver. 8.2.10 under the GTR+G nucleotide substitution model and 1,000 rapid bootstraps. The BI analysis was performed in MrBayes version 3.2 (Ronquist et al. 2012). MrModeltest version 2.2 (Nylander 2004) was used for BI analysis to determine a best-fit model of nucleotide substitution. From a random starting tree, the BI analysis was run for 10 million generations and the trees were saved to a file every 1,000 generations. Posterior probabilities were approximated by sampling trees using a variant of the Markov Chain Monte Carlo (MCMC) method. The first 1,000 trees were discarded as “burn-in” and a majority-rule consensus tree was calculated based upon the remaining 9,000 trees resulting from Tracer 1.4 analysis (Drummond and Rambaut 2007).

## Results

### Plant morphology

**Figure 1.**
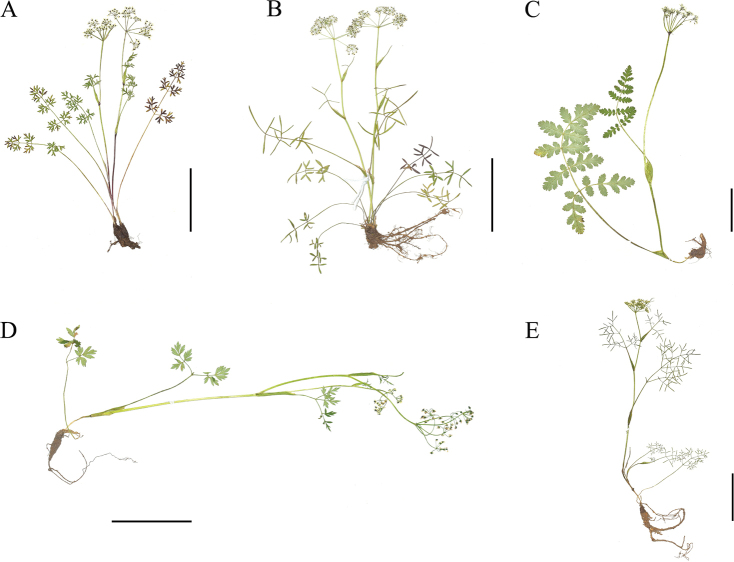
Specimens of *Sinocarum***A***S. coloratum***B***S. cruciatum***C***S. filicinum***D***S. schizopetalum***E***S. vaginatum*. Scale bars: 5 cm.

The plant morphological characteristics of *Sinocarum* species are shown in Table 3 and Fig. 1, and we can know that *Sinocarum coloratum* typically possesses purplish stems, oblong-ovate sheaths, lanceolate ultimate segments of blades and white petals (Fig. 1A). *S. cruciatum* generally has torulose roots, subequal rays, triangular in outline and ternate-1–2-pinnate basal leaves, and reduced upward to 1-pinnate or 3-lobed cauline leaves (Fig. 1B). *S. filicinum* develops broadly ovate sheaths, linear-lanceolate bracts and bracteoles, triangular in outline and 2-pinnate blades, oblong-ovate blade ultimate segments with serrated margins, and sparsely pubescent petioles, rachides and the abaxial surface of segments (Fig. 1C). *S. schizopetalum* typically has conic taproot, broad-lanceolate sheaths, triangular in outline and ternate-1–2-pinnate blades with oblong-lanceolate ultimate segments, and white or violet petals (Fig. 1D). *S. vaginatum* generally possess ovate sheaths, unequal rays, entire petals with acute apexes, triangular and ternate-2–3-pinnate blades with elongate-linear ultimate segments, and reduced upwards to 1–2-pinnate cauline leaves (Fig. 1E).

**Table 3.** Comparison of plant morphological characteristics of *Sinocarum*.

Characteristics	*S. coloratum*	*S. cruciatum*	*S. filicinum*	*S. schizopetalum*	*S. vaginatum*
Root	taproot elongate, thickened at apex, branched	rootstock short, thick; roots torulose	taproot elongate, stout, often branched	taproot conic	taproot elongate, thick, often branched
Stems	characteristically purplish	green	green	green	green
Basal petioles	2–10 cm	5–7 cm	8–15 cm, sparsely pubescent	5–8 cm	5–18 cm
Sheath	oblong-ovate	oblong-ovate	broadly ovate	broadly lanceolate	ovate
Basal leaves	blade ovate-lanceolate in outline, 1–2-pinnate; pinnae 4–5 pairs	triangular in outline, ternate-1–2-pinnate; pinnae 3–5 pairs	triangular in outline, 2-pinnate; pinnae 3–7 pairs	triangular in outline, ternate to 1- or 2-pinnate	triangular in outline, ternate-2–3-pinnate; pinnae 4–6 pairs
Ultimate segments of blade	linear-lanceolate	linear-lanceolate	oblong-ovate, margins serrate, abaxially sparsely pubescent along veins	oblong-lanceolate	elongate-linear
Cauline leaves	similar to the basal leaves	elongate-linear, reduced upwards becoming 1-pinnate or 3-lobed	similar to the basal leaves; upper leaves 1-pinnate	similar to the basal leaves	elongate-linear, 1–2-pinnate, reduced upwards
Bracts	absent or occasionally 1–2, linear	absent, occasionally 1	1–4, linear-lanceolate	absent, occasionally 1, linear-lanceolate	absent or occasionally 1
Bracteoles	absent, rarely 1, linear	absent, occasionally 1	5–8, linear-lanceolate	3–5, linear-lanceolate	absent
Rays	5–8(–12), unequal	4–7(–10), subequal	2–8, 1–3 cm, unequal	(3–)5–6(–8), unequal	10–12, unequal
Petals	ovate or broadly obovate, apex usually entire, occasionally 2–3-lobed, white	oblong-ovate or broadly obovate, apex obtuse to subacute, greenish-white	ovate or broadly obovate, apex subacute, white	apex palmately 3–4-lobed, lobes lanceolate or oblanceolate, white or violet	oblong-ovate or broadly obovate, apex subacute, white

### Fruit morphology and anatomy

**Figure 2.**
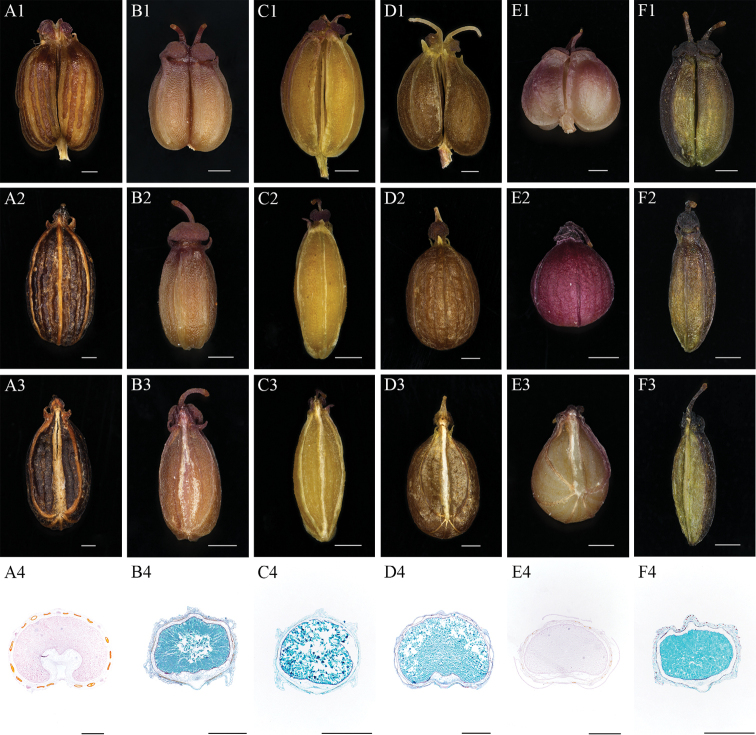
Morphological and anatomical characteristics of mature fruit **A***Meeboldia yunnanensis***B***Sinocarum coloratum***C***S. cruciatum***D***S. filicinum***E***S. schizopetalum***F***S. vaginatum*. Scale bars: 0.5 mm.

The fruit morphological and anatomical characteristics of *Sinocarum* species and *Meeboldia yunnanensis* were studied. The results are shown in Table 4 and Fig. 2. We found that the mature fruits of *Meeboldia yunnanensis* are ovoid with 5 filiform inconspicuous ribs, 2–3 vittae in each furrow and 4 on commissure, semicircle transection of mericarp and cordate concave endosperm concrescence (Fig. 2A1–A4). The ovoid mature fruits of *S. coloratum* have 1–3 vittae in each furrow and 2–4 on commissure, sub-pentagon transection and flat endosperm concrescence (Fig. 2B1–B4). *S. cruciatum* typically has oblong-ovoid fruits with 5 filiform ribs, 1–3 vittae in each furrow and 2–4 on commissure, and sub-pentagon transection and flat endosperm concrescence (Fig. 2C1–C4). The mature fruits of *S. filicinum* are generally ovoid with slightly constricted apex, obscure ribs, 2–3 vittae in each furrow and 4 on commissure, semicircle transection and sub-cordate concave endosperm concrescence (Fig. 2D1–D4). The mature fruits of *S. schizopetalum* are broad-ovoid with obscure ribs, 1–3 vittae in each furrow and 2 on commissure, semicircular transection of mericarp, and slightly concave endosperm concrescence (Fig. 2E1–E4). The mature fruits of *S. vaginatum* are typically oblong-ovoid with 1–3 vittae in each furrow and 2–4 on commissure, sub-pentagon transection and flat endosperm concrescence (Fig. 2F1–F4).

**Table 4.** Mature fruit morphological and anatomical characteristics of *Sinocarum* species and *Meeboldia yunnanensis*.

Species	Fruit shape		Transection	Endosperm concrescence	Development degree of ribs	Vittae number	
	L × W (mm)	Shape				Furrow	Commissure
*Meeboldia yunnanensis*	4.08 × 2.35	ovoid	semicircle	cordate concave	unobvious	2–3	4
*S. coloratum*	2.09 × 1.16	ovoid	sub-pentagon	flat	obvious	1–3	2–4
*S. cruciatum*	2.41 × 0.96	oblong-ovoid	sub-pentagon	flat	obvious	1–3	2–4
*S. filicinum*	2.81 × 1.97	ovoid	semicircle	sub-cordate concave	unobvious	2–3	4
*S. schizopetalum*	1.43 × 1.36	broad-ovoid	semicircle	slightly concave	unobvious	1–3	2
*S. vaginatum*	2.28 × 0.90	oblong-ovoid	sub-pentagon	flat	obvious	1–3	2–4

### Palynology

**Figure 3.**
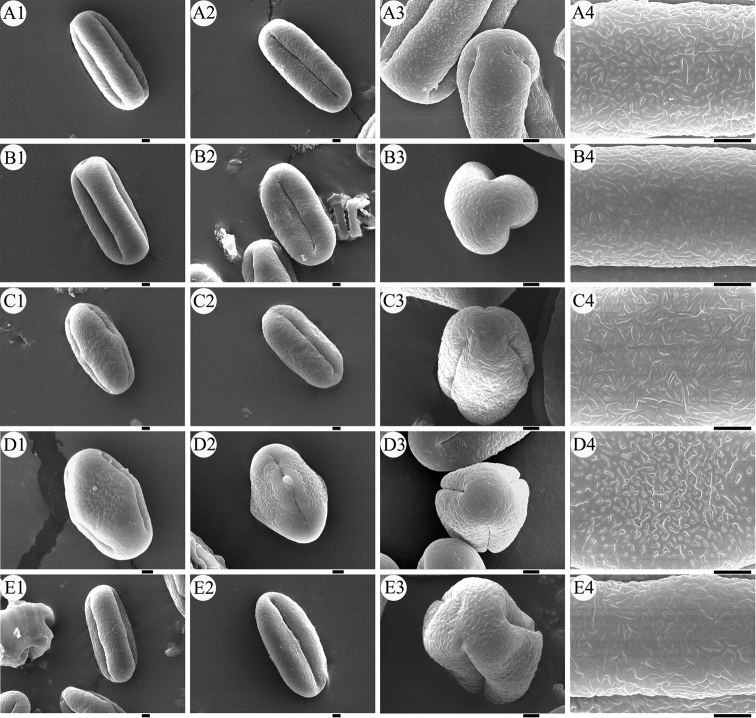
Pollen morphology in scanning electron microscope (SEM) **A***Sinocarum coloratum***B***S. cruciatum***C***S. filicinum***D***S. schizopetalum***E***S. vaginatum*. Scale bars: 2 μm.

The pollen morphology of the five *Sinocarum* species was studied by SEM, as shown in Table 5 and Fig. 3. The average ratio of the polar axis to the equatorial axis (P/E) of the pollen grains of *S. coloratum*, *S. cruciatum* and *S. vaginatum* is greater than 2, and the average size index was greater than 19. The pollen grains of these three species are super-rectangular in equatorial view, trilobate circular in polar view (Fig. 3A1–A3, B1–B3, E1–E3). The exine ornamentation of the polar area is cerebroid with a few perforations, and the equatorial area is cerebro reticulate (Fig. 3A3–A4, B3–B4, E3–E4). The pollen grains of *S. filicinum* are super-rectangular in equatorial view, trilobate circular in polar view (Fig. 3C1–C3). The exine ornamentation of the polar area is striate reticulate with a few perforations, and the equatorial area is cerebro reticulate (Fig. 3C3–C4). Compared with other *Sinocarum* species, the pollen size of *S. schizopetalum* is smaller, and its size index is 15.11(14.15~16.62). And its pollen grains are sub-rhomboidal in equatorial view, obtuse triangled in polar view (Fig. 3D1–D3). The exine ornamentation of the polar area is cerebroid, and the equatorial area is pitted reticulate (Fig. 3D3–D4).

**Table 5.** Pollen morphology of *Sinocarum* species in scanning electron microscope (SEM).

Species	Type	Shape		Size(μm)	Polar axis/Equatorial axis(P/E)	Size index ($\sqrt{P\times E}$)	Exine ornamentation	
		Equatorial view	Polar view				Equatorial area	Polar area
*S. coloratum*	super-rectangular	super-rectangular	trilobate circular	(27.20~32.90)30.36× (11.72~13.95)12.78	2.38 (2.16~2.59)	19.69 (18.51~20.00)	cerebro reticulate	cerebroid with a few perforations
*S. cruciatum*	super-rectangular	super-rectangular	trilobate circular	(26.07~32.15)28.84× (11.42~15.33)13.24	2.19 (1.80~2.56)	19.52 (17.25~20.92)	cerebro reticulate	cerebroid with a few perforations
*S. filicinum*	super-rectangular	super-rectangular	trilobate circular	(22.46~27.08)24.95× (11.12~13.57)12.56	1.99 (1.66~2.28)	17.69 (15.80~18.68)	cerebro reticulate	striate reticulate with a few perforations
*S. schizopetalum*	sub-rhomboidal	sub-rhomboidal	obtuse triangle	(18.27~20.81)19.61× (10.40~13.31)11.66	1.69 (1.56~1.92)	15.11 (14.15~16.62)	pitted reticulate	cerebroid
*S. vaginatum*	super-rectangular	super-rectangular	trilobate circular	(25.83~29.30)27.44× (10.92~14.62)13.58	2.03 (1.82~2.37)	19.29 (16.79~20.20)	cerebro reticulate	cerebroid with a few perforations

### Sequence characteristics

The characteristics of the three DNA regions are summarized in Table 6. These results indicated that the aligned length of the background tree using 59 ITS sequences from Apiaceae was 472, containing 73.94% average sequence divergence and 276 parsimony informative characters. In addition, average sequence divergence of ITS was more variable (60.50%; 56.76%) than cpDNA (16.76%; 13.06%) across the *Acronema* clade, East Asia clade and their outgroups. Overall, ITS was more variable than cpDNA, with greater average sequence divergence and more parsimony informative characters.

**Table 6.** Statistical summary for ITS and the cpDNA regions used to infer phylogenetic relationships of *Sinocarum*.

		Apioideae	*Acronema* clade		East Asia clade	
		ITS	ITS	cpDNA(*rpl16*+*rps16*)	ITS	cpDNA(*rpl16*+*rps16*)
No. of accessions		59	44	45	34	52
Aligned length		472	476	1897	444	2128
No. variable characters		349 (73.94%)	288 (60.50%)	318 (16.76%)	252 (56.76%)	278 (13.06%)
No. parsimony informative characters		276 (58.47%)	205 (43.07%)	143 (7.54%)	184 (41.44%)	163 (7.66%)
Model	ML	GTR+G	GTR+G	GTR+G	GTR+G	GTR+G
	BI	–	GTR+G	GTR+I+G	GTR+G	GTR+G

ITS, internal transcribed spacer; ML, maximum likelihood; BI, Bayesian Inference.

### Phylogenetic analyses

#### ITS trees (Figs 4, 5A, 6A)

**Figure 4.**
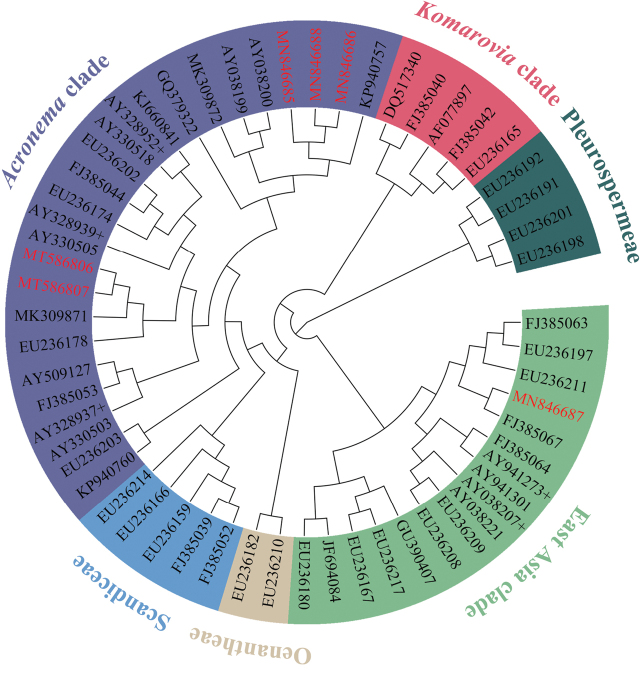
Phylogenetic relationships inferred from maximum likelihood (ML) analysis of 59 nrDNAITS sequences from Apiaceae subfamily Apioideae. The tree is rooted with Pleurospermeae. The names of the clades are identified by Zhou et al. (2008, 2009) and Downie et al. (2010).

**Figure 5.**
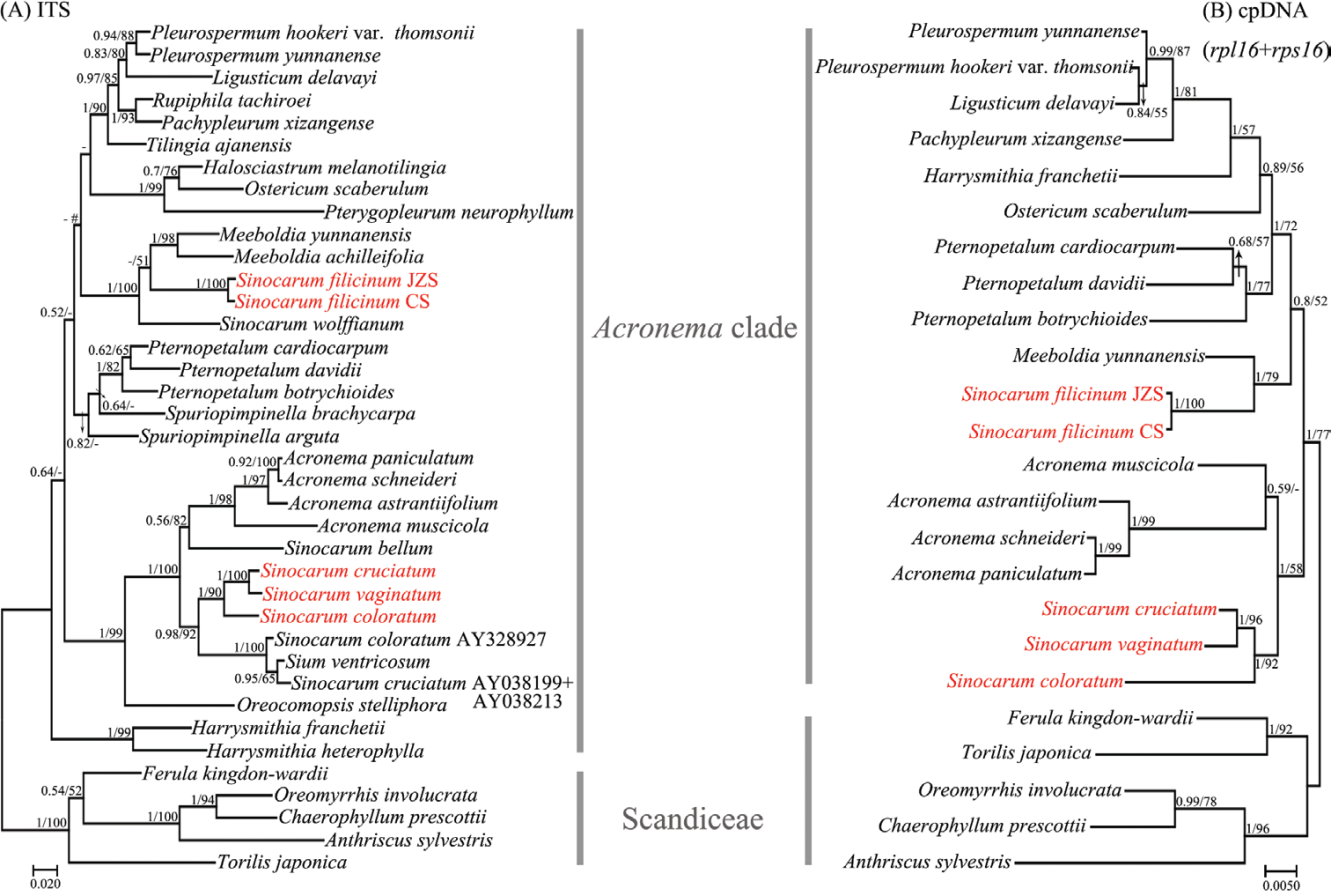
Bayesian 50% strict consensus trees of 44 nrDNAITS sequences (**A**) and 45 combined cpDNA*rpl16* and *rps16* intron sequences (**B**) from *Acronema* clade and outgroups. Values on the branches indicate its support (Bayesian posterior probability/ bootstrap value). Those nodes not occurring in the ML strict consensus tree are indicated by pound symbols (#). Short line denotes values < 50%. The tree is rooted with Scandiceae. The names of the clades are identified by Zhou et al. (2008, 2009) and Downie et al. (2010).

**Figure 6.**
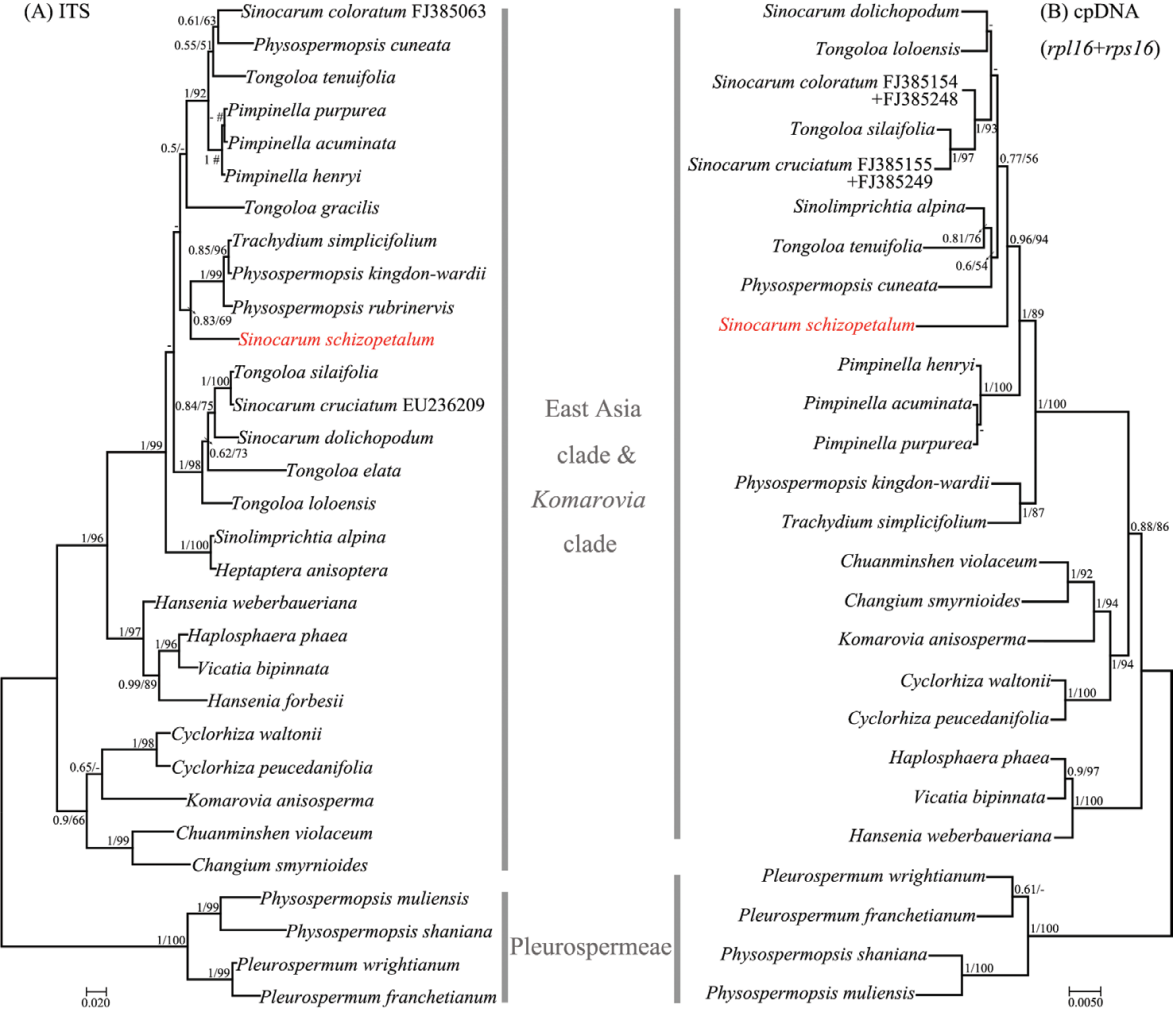
Bayesian 50% strict consensus tree of 34 nrDNAITS sequences (**A**) and 52 combined cpDNA*rpl16* and *rps16* intron sequences (**B**) from East Asia clade & *Komarovia* clade and outgroups. Values on the branches indicate its support (Bayesian posterior probability/ bootstrap value). Those nodes not occurring in the ML strict consensus tree are indicated by pound symbols (#). Short line denotes values < 50%. The tree is rooted with Pleurospermeae. The names of the clades are identified by Zhou et al. (2008, 2009) and Downie et al. (2010).

After phylogenetic analyses with comprehensive sequence data, we confirmed that the collected *Sinocarum* materials were from a polyphyletic group and fell into two different clades (i.e. *Acronema* clade, East Asia clade) according to the ITS tree inferred by ML approach (Fig. 4). The ITS trees of *Acronema* clade and its outgroups (Fig. 5A) inferred by ML and BI approaches were inconsistent for subclade topology. The ITS tree demonstrated again that *Sinocarum* was a polyphyletic group. Additionally, it indicated that the collected *Sinocarum* materials (*S. coloratum*, *S. cruciatum* and *S. vaginatum*), together with two previously sequenced *Sinocarum* species (*S. coloratum*AY328927; *S. cruciatum*AY038199; AY038213) and *Sium ventricosum* (H. Boissieu) Li S. Wang & M. F. Watson (2016: 266) (AY038200; AY038214) constituted a strongly supported group (Bayesian Inference (BI)–posterior probability (PP)=0.98/Maximum Likelihood (ML)–bootstrap value (BS)=92%), as sister group to four species of *Acronema* and *Sinocarum bellum* (C. B. Clarke) Pimenov & Kljuykov (2006: 122) (PP/BS = 1/100%). A newly recognized but weakly supported subclade in the ITS trees encompassed four species of *Acronema* and *Sinocarum bellum* (PP/BS = 0.56/82%). Of three *Sinocarum* species we collected, *S. cruciatum* was allied closer with *S. vaginatum* than *S. coloratum* (PP/BS = 1/100%). Two populations of *S. filicinum*, two species of *Meeboldia* H. Wolff (1924: 313) and *S. wolffianum* (Fedde ex H. Wolff) P. K. Mukh. & Constance (1991: 42) formed a strongly supported subclade (PP/BS = 1/100%), and *S. filicinum* had a closer relationship with *Meeboldia.* The ITS trees of the East Asia clade and its outgroups (Fig. 6A) inferred by ML and BI approaches were inconsistent for *Pimpinella* L. (1753: 263) subclade topology. In addition, the trees indicated that *S. schizopetalum* was allied distantly with other collected *Sinocarum* species, and closely related to *Trachydium*Lindley (1835: 232) subclade (PP/BS = 0.83/69%) in the strongly supported East Asia clade (PP/BS = 1/96%).

#### cpDNA trees (Figs 5B, 6B)

The cpDNA trees of *Acronema* clade and its outgroups (Fig. 5B) inferred by ML and BI approaches had consistent topologies. The cpDNA trees indicated that the generic type of *Sinocarum*, *S. coloratum*, together with *S. cruciatum* and *S. vaginatum* constituted a supported monophyletic group (PP/BS = 1/92%) as sister group to *Acronema* (PP/BS = 1/58%). Two populations of *S. filicinum* allied powerfully with the genus *Meeboldia* (PP/BS = 1/79%). The cpDNA trees of the East Asia clade and its outgroups (Fig. 6B) inferred by ML and BI approaches had consistent topologies and indicated that the position of *S. schizopetalum* differed from the ITS tree and was located in the East Asia clade.

## Discussion

### Morphology

We have studied the plant morphology, fruit morphological and anatomical characteristics, and palynology of five species of *Sinocarum*, and perfected the mature fruit characteristics of these species. Through the analysis of comprehensive morphological data, the five *Sinocarum* species can be divided into three groups. Group 1 includes *S. coloratum*, *S. cruciatum* and *S. vaginatum*. They are characterized by slender and glabrous plants, usually ovate or oblong-ovate sheath, mostly absent bracts and bracteoles, typically entire petals, ovoid or oblong-ovoid mature fruit with 1–3 vittae in each furrow and 2–4 on commissure, sub-pentagon transection and flat endosperm concrescence. And the pollen grains of these three species are super-rectangular in equatorial view, trilobate circular in polar view. Group 2 includes *S. filicinum*, whose morphological characteristics were significantly different from those of other *Sinocarum* species we collected, and the key identification features were the linear-lanceolate bracts and bracteoles, oblong-ovate blade ultimate segments with serration on the margins, sparsely pubescent petioles, rachides and the abaxial surface of segments. Group 3 includes *S. schizopetalum*, whose most prominent features are apex palmately 3–4-lobed and white or violet petals, broad-ovoid mature fruits and sub-rhomboidal pollen. Among them, petal characteristics are very special in the whole genus *Sinocarum*. It is concluded that plant morphology, fruit morphological and anatomical characteristics, and palynology have important taxonomic significance.

### Phylogenetic placement of *Sinocarum*

Previous studies have shown that *Sinocarum* is not a monophyletic group and the phylogenetic placement remains unclear (Valiejo-Roman et al. 2002; Zhou et al. 2008; Zhou et al. 2009; Downie et al. 2010). *S. coloratum* is the generic type of *Sinocarum*, its phylogenetic placement represents the phylogenetic placement of *Sinocarum.* In this study, we confirmed that *Sinocarum* is not a monophyletic group and used three sequences of *S. coloratum* (MN846685, AY328927, FJ385063), our sequenced specimen and two downloaded sequences, to determine the phylogenetic placement of *Sinocarum*. We found that one of the downloaded (FJ385063) was located in the East Asia clade, while our accession and the other downloaded accession were located in the *Acronema* clade but these two were not clustered together. The results of our field investigation, morphological study and specimen verification of *S. coloratum* obtained from the type locality (Mt. Yulong) showed that our collected material is highly consistent with the type specimen and the original literature description of *S. coloratum*. In conclusion, the true phylogenetic placement of *Sinocarum* is within the *Acronema* clade and the genus has a close affinity with *Acronema*.

### The relationship between *Sinocarum* and *Acronema*

This study’s phylogeny results indicated that there was a close and complex relationship between *Sinocarum* and *Acronema*. Fusiform or elongate roots and apex slightly obtuse or rarely lobed petals are easily recognizable characteristics of *Sinocarum*, and an apex long-linear or long-aristate petal is the most prominent feature of *Acronema*. In fact, within each genus there are species that deviate in one or more morphological characteristics from the typical and the generic boundaries are blurred with a few species being easily confused as belonging to the other genus (Watson 1996; Watson et al. 2004; Pu et al. 2005). For example, *S. cruciatum* has torulose roots and several species of the genus *Acronema*, *A. chienii* R. H. Shan & S. L. Liou (1980: 197), *A. chinense* H. Wolff (1926: 309) have apex acute or obtuse-acute petals, characteristics typically observed in the other genus. Through a literature review, field investigation, morphological study and specimen examination, we found that the plants of *Sinocarum* and *Acronema* are all slender. In addition, *Sinocarum* and *Acronema* are both distributed in the high-elevation Sino-Himalayan region from Nepal to SW China. The habitat of the two genera is extremely similar as they are distributed in the humid environment of rock crevices, alpine meadows or shady forests. These conditions provide further evidence for the close and complex affinity between the two genera. This study and others have provided cumulative evidence to reduce phylogenetic uncertainty. Despite recent collections, the range of materials for the two genera is still limited and more field specimens will be required to provide a comprehensive revision of the phylogeny of *Sinocarum* and *Acronema* across their geographic range.

### Infrageneric relationships

Our ITS and cpDNA trees showed that *S. coloratum* (generic type), *S. cruciatum* and *S. vaginatum* clustered together, but *S. cruciatum* had a closer relationship with *S. vaginatum*, which was consistent with the results of morphological study. The closer relationship between *S. cruciatum* and *S. vaginatum* is supported by the ultimate segments of their blades being more slender than other *Sinocarum* species and forming a group of narrow-leaved taxa. However, *S. vaginatum* develops elongate-linear ultimate segments of basal leaves and cauline leaves, and more rays, about 10–12. Whereas *S. cruciatum* has subequal rays and torulose roots. These two species are recognizable by these major features. In addition, *S. cruciatum* and *S. vaginatum* were both collected from the Dali range, Dali, Yunnan, overlapping in their ranges. Consequently, the morphological evidence and geographical distribution are consistent with the phylogenetic analysis results.

*Sinocarum filicinum* H. Wolff (1929: 182) was originally described by Wolff (1929) based on G. Forrest n. 6863, 11691, 7230, and obtained from the eastern flank of the Dali Range in Yunnan. Since the description of *S. schizopetalum*, its phylogenetic placement has been controversial. Franchet (1894) originally described it as a new species as *Carum schizopetalum* Franch., and Wolff (1927) transferred it to *Sinocarum*, later Wu Zhengyi (1984) transferred this species to *Dactylaea* H. Wolff (1930: 304) as *Dactylaea schizopetala* (Franch.) Wu Zhengyi (1984: 910). Pu et al. (2005) accepted Wolff’s view in the description of *Sinocarum* in *Flora of China*. Pimenov (2017) also accepted Wolff’s view that this species belongs to *Sinocarum* after studying the specimens of Apiaceae, especially the type specimens. In addition, no molecular phylogenetic studies have been carried out on *S. filicinum* and *S. schizopetalum*.

The results showed that the two populations of *S. filicinum* were not related to *S. coloratum* (*Sinocaurm* generic type) and allied most closely with the genus *Meeboldia*, according to the ITS and cpDNA trees. Our morphological results indicated that the morphological characteristics of *S. filicinum* are distinct from the three other *Sinocarum* species in the *Acronema* clade that we collected and are very consistent with the characteristics of *Meeboldia*. Among them, fruit characteristics play a key role of subfamily Apioideae classification (Kljuykov et al. 2004; Lyskov et al. 2017; Guo et al. 2018; Jia et al. 2019). And the fruit characteristics of *S. filicinum* are similar to those of *Meeboldia yunnanensis*, they are all ovoid, with 5 filiform inconspicuous ribs, 2–3 vittae in each furrow and 4 on commissure, semicircle transection of mericarp and cordate concave or sub-cordate endosperm concrescence (Fig. 2A, D). The molecular data and morphological evidence indicated that *S. filicinum* is closely related to *Meeboldia* and should be isolated from *Sinocarum*, but due to the lack of comprehensive samples, the phylogenetic placement will not be revised at present.

Our phylogenetic results showed that *S. schizopetalum* was distantly related to the other *Sinocarum* species we collected (*S. coloratum*, *S. cruciatum*, *S. filicinum* and *S. vaginatum*). We found that the exact phylogenetic placement of *S. schizopetalum* was inconsistent between the ITS tree and the cpDNA tree, but was nevertheless located in the East Asia clade. Morphologically, *S. schizopetalum* has apex palmately 3–4-lobed petals, broad-ovoid mature fruits and sub-rhomboidal pollen, and these features are clearly distinct from other species of *Sinocarum*. Unlike the other studied *Sinocarum* species, the plant morphology, fruit and pollen morphology of *S. schizopetalum* are more similar to species of the East Asia clade. According to the results of phylogeny and morphology studies, it is suggested *S. schizopetalum* should be isolated from *Sinocarum*. However, due to the complex taxonomic problems among genera in the East Asia clade, the phylogeny of *S. schizopetalum* cannot be resolved. Thus, *S. schizopetalum* needs revision pending expanded sampling and phylogenetic analyses to include more East Asia clade species from the Sino-Himalayan region.
